# CFCPred: Advancing circRNAs‐Encoded Peptides Prediction Through Cluster Purity‐Guided Resampling and Fuzzy Voting

**DOI:** 10.1049/syb2.70084

**Published:** 2026-08-07

**Authors:** Siyuan Zhao, Bin Yu, Lingling Liu

**Affiliations:** ^1^ School of Information Engineering Tianjin University of Commerce Tianjin China

**Keywords:** biology computing, data mining, genomics, pattern classification

## Abstract

Circular RNAs (circRNAs) have recently been shown to possess coding potential via a cap‐independent translation mechanism, challenging their traditional classification as noncoding RNAs and implicating it directly in the pathogenesis of various diseases. Despite growing evidence of the functional roles of circRNAs‐encoded peptides (circPEPs) in disease mechanisms, computational methods for predicting these peptides remain underdeveloped. In this study, we introduce CFCPred, a novel tool for circPEPs prediction that combines cluster purity‐guided resampling with fuzzy voting (FV) within a traditional machine learning framework. To address the scarcity of known circPEPs, we employ a resampling strategy that mitigates class‐imbalance in the training data by generating synthetic samples based on relationships among individual samples, their nearest neighbours and their cluster assignments. Additionally, to tackle class overlap in the testing data, FV enables adaptive model selection and dynamic weight adjustment across the ensemble. This adaptive ensemble integrates predictions from multiple base models to produce a robust consensus classification. Experimental validation across multiple independent datasets demonstrates that CFCPred achieves strong predictive performance. Overall, the tool provides a computational framework that can accelerate research into circPEPs and their roles in disease.

## Graphical abstract

CFCPred is a novel circPEPs prediction tool that integrates cluster purity‐guided resampling and fuzzy voting within a traditional machine learning framework. A resampling technology is introduced to mitigate class‐imbalance in training samples by synthesising samples through an analysis of the relationships among individual samples, their nearest neighbours and their cluster assignments. To address the challenge of class overlap in testing samples, fuzzy voting performs adaptive model selection and dynamic weight adjustment for the ensemble. This adaptive ensemble fuses the outputs of diverse base models to arrive at a robust consensus classification.

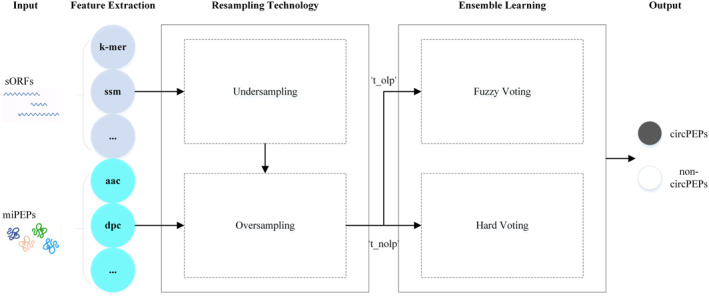

## 1 Introduction

Circular RNAs (circRNAs) are characterised by covalently closed loop structures, lacking the canonical 5′ cap and 3′ poly(A) tail [1]. Traditionally recognised as molecular sponges and regulators of parental gene expression, circRNAs have been broadly implicated in disease pathogenesis [2, 3]. Notably, recent studies have revealed that circRNAs also possess intrinsic coding potential [4]. They can undergo noncanonical translation, primarily mediated by internal ribosome entry sites and N6‐methyladenosine (m6A) modifications, to express their encoded open reading frames (ORFs), referred to as circRNAs‐derived ORFs (cORFs).

Emerging evidence indicates that circPEPs play significant functional roles in disease pathogenesis [5, 6, 7]. For example, circZKSCAN1‐derived circZKSaa suppresses hepatocellular carcinoma proliferation by interacting with FBXW7 to inhibit mTOR ubiquitination [5]. Similarly, PINT87aa, encoded by circLINC‐PINT, promotes cellular senescence and inhibits cell growth while suppressing mitophagy through its interaction with FOXM1 and the subsequent downregulation of its target gene, PHB2 [6]. In contrast, circPPP1R12A‐73aa enhances the proliferation and viability of NSCLC cells, suppresses apoptosis and activates the AKT signalling pathway, although it does not significantly affect cell invasion or migration [7]. Collectively, these findings highlight the translational relevance of circPEPs in oncology and helps to expand our understanding of the functional repertoire of the eukaryotic genome, providing novel mechanistic insights with potential implications for therapeutic and vaccine development.

Although biological assays such as qRT‐PCR and western blotting have been used to validate circPEPs, these approaches are often limited by high cost, time‐consuming procedures and increased uncertainty when applied to novel biological contexts [8]. Although computational methods for identifying cORFs have emerged, many existing approaches are overly complex and difficult to generalise [9, 10, 11]. Several coding potential prediction tools, including CRITICA [12], CPC2 [13] and PhyloCSF [14], utilise sequence alignment to distinguish long noncoding RNAs (lncRNAs) from messenger RNAs (mRNAs). These tools can also predict coding sequences, as the primary distinction between mRNAs and lncRNAs lies in their coding potential.

Several computational tools have been developed to identify small RNAs with coding potential. CPPred, proposed by Tong et al. [15], employs a support vector machine (SVM) to predict coding RNAs using a combination of CTD features and sequence‐based features derived from CPAT and CPC2. DeepCPP [16] and CPE‐SLDI [17] represent enhancements of CPPred. Zhang et al. [16] developed DeepCPP, a convolutional neural network‐based model trained and tested on newly constructed small ORFs datasets spanning multiple species, to evaluate the coding potential of lncRNAs. Chen et al. [17] introduced CPE‐SLDI, which specifically addresses class‐imbalance in small ORFs‐containing RNAs using oversampling techniques. csORF‐finder [18], developed by Zhang et al., is a region‐specific tool that combines an efficient CapsNet architecture with a light gradient boosting machine to distinguish small ORFs across different genomic regions. In addition, several tools have been designed to predict peptides encoded by small ORFs. SORFPP, developed by Feng et al. [19], is an ensemble prediction tool that integrates ESM‐2 embeddings (analysed via self‐attention) with sparsity‐processed traditional encodings (via CatBoost) through logistic regression to accurately identify sORFs‐encoded peptides. pLM4PEP, proposed by Yue et al. [20], is a micro RNAs‐encoded peptides prediction tool based on protein language models. It performs classification using logistic regression on eight integrated features, including two scales of ESM2 embeddings and six manually curated features. Experimental validation demonstrated the strong predictive performance of pLM4PEP across multiple peptide types.

Most existing tools perform indirect prediction of circPEPs by translating nucleotide sequences into amino acid information. However, their effectiveness is limited by two major challenges. First, the training data are highly heterogeneous in length, with shorter sequences often producing sparse feature representations after encoding. The substantial differences in length and composition between standard ORFs (producing proteins) and cORFs (producing circPEPs) complicate the development of predictive models that generalise well. Because machine learning algorithms are fundamentally benchmarked against their training data, models risk capturing features specific to conventional ORFs, potentially failing to generalise to the unique physicochemical and structural space of circPEPs or learning spurious correlations that do not hold in practice. Second, the scarcity of known circPEPs creates severe class‐imbalance. Class‐imbalance, a common challenge in machine learning, occurs when training datasets have an unequal distribution of categories, making model training more difficult and reducing predictive reliability.

In this study, we propose CFCPred, a novel circPEPs prediction tool that integrates cluster purity‐guided resampling with fuzzy voting (FV). Multiple feature descriptors are extracted separately from circPEPs and cORFs. To address the severe class‐imbalance caused by the scarcity of known circPEPs, we introduce a resampling technique called CluPg, which generates synthetic samples by analysing the relationships among individual samples, their nearest neighbours and their cluster assignments. Specifically, cORFs‐associated features are incorporated as prior knowledge during the undersampling stage of CluPg, whereas circPEPs‐associated features guide its oversampling stage. Using the balanced dataset enriched with circPEPs features, several machine learning models are then trained. To further tackle class overlap in testing samples, FV is applied for adaptive model selection and dynamic weight adjustment within the ensemble. This adaptive ensemble combines predictions from multiple base models to produce a robust consensus classification. Experimental results demonstrate that CFCPred significantly outperforms current state‐of‐the‐art tools across independent testing sets. The Python scripts for CFCPred are available at https://github.com/zzssyy/CFCPred.

## 2 Materials and Methods

### 2.1 Structure of Our Research for circPEPs Prediction

**FIGURE 1:**
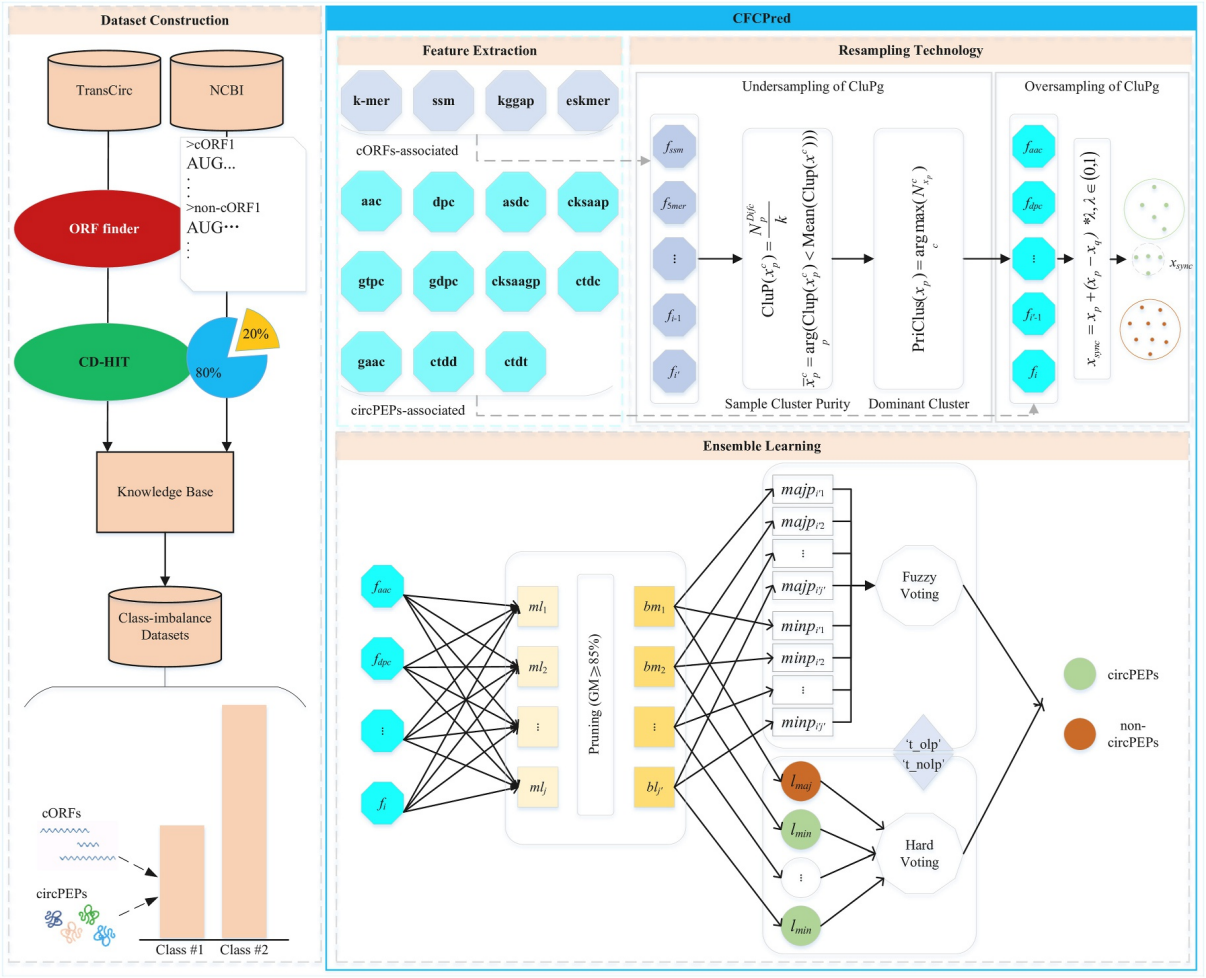
Structure of our research for circPEPs prediction. It consists of dataset construction and CFCPred. Dataset construction illustrates the general process of constructing the dataset. CFCPred is used to predict circRNAs‐encoded peptides.

The structure of our study consists of two main components: dataset construction and CFCPred. CFCPred is designed to predict whether a given input sequence can be confidently classified as a circPEP. The framework of CFCPred comprises three key modules: feature extraction, resampling and ensemble learning. A schematic overview of our approach for circPEPs prediction is presented in Figure 1.

### 2.2 Dataset Construction

Sequences with coding potential derived from circRNAs are obtained from the TransCirc database (https://www.biosino.org/transcirc/) [21]. These sequences span multiple species, including *Homo sapiens* (*H. sapiens*), *Mus musculus* (*M. musculus*), *Rattus norvegicus* (*R. norvegicus*) and *Drosophila melanogaster* (*D. melanogaster*). cORFs derived from these sequences are identified using ORF Finder [22]. Noncoding RNAs (ncRNAs) sequences are retrieved from the NCBI database (https://www.ncbi.nlm.nih.gov/) [22], and noncORFs are also extracted using ORF Finder. Amino acid sequences corresponding to circPEPs and noncircPEPs are generated based on the nucleotide‐to‐amino acid mapping. The lengths of these sequences are predominantly between 30 and 200 amino acids, which defined the candidate dataset for further analysis.

Sequence redundancy is removed to screen candidate data. Sequences with more than 80% similarity are filtered out using the CD‐HIT tool [23]. Using the training–validation set as a reference, the independent testing set is initially filtered using CD‐HIT‐2D with a 50% threshold to remove sequences exhibiting ≥ 50% similarity to any reference sequence [23]. Subsequently, BLAST is employed to further eliminate independent testing sequences sharing > 80% identity with sequences in the training–validation set [22]. The knowledge base is constructed using codon information and sequence length, with AUG designated as the start codon. Positive and negative samples are selected to ensure comparable sequence lengths. This procedure generated the final dataset, which is divided into Dataset1, Dataset2 and Dataset3 for training, validation and testing purposes. Because of the unequal numbers of positive and negative samples, the datasets are inherently imbalanced. Dataset1 is used as the training–validation set, whereas Dataset2 and Dataset3 serve as independent‐testing sets. Dataset3 includes sequences from additional species, resulting in substantial compositional variation. Detailed information on the datasets is provided in Table 1.

**TABLE 1:** Detail of datasets used to predict circPEPs.

Dataset	Type	Species	#Minority class (positive) sample	#Majority class (negative) sample	Imbalanced ratio
Dataset1	Training–validation set	*Homo sapiens*	63	315	—
		—	63	315	5.0
Dataset2	Independent‐testing set	*H.sapiens*	30	60	—
		*Mus musculus*	40	80	—
		—	70	140	2.0
Dataset3	Independent‐testing set	*M.musculus*	31	310	—
		*Rattus norvegicus*	7	70	—
		*Drosophila melanogaster*	2	20	—
		—	40	400	10.0

*Note:* # Represents the number of samples.

### 2.3 Feature Extraction

Domain‐specific feature representation is derived through knowledge‐guided extraction processes. Some feature descriptors, including AAC (*de* = 20*, V*_*aac*_), DPC (*de* = 400*, V*_*dpc*_), ASDC (*de* = 400*, V*_*asdc*_), C1SAAP (*de* = 400*, V*_*c*1*saap*_), C2SAAP (*de* = 400*, V*_*c*2*saap*_), C3SAAP (*de* = 400*, V*_*c*3*saap*_), C1SAAGP (*de* = 400*, V*_*c*1*saagp*_), C2SAAGP (*de* = 400*, V*_*c*2*saagp*_), C3SAAGP (*de* = 400*, V*_*c*3*saagp*_), GAAC (*de* = 5*, V*_*gaac*_), GDPC (*de* = 25*, V*_*gdpc*_), GTPC (*de* = 125*, V*_*gtpc*_), CTDC (*de* = 39*, V*_*ctdc*_), CTDT (*de* = 39*, V*_*ctdt*_) and CTDD (*de* = 195*, V*_*ctdd*_), are extracted from circPEPs or noncircPEPs and are recorded as cirPEPs‐associated feature descriptors. Other feature descriptors, including ES3MER (*de* = 64*, V*_*es*3*mer*_), ES4MER (*de* = 256*, V*_*es*4*mer*_), ES5MER (*de* = 1024*, V*_*es*5*mer*_), 5mer (*de* = 1364*, V*_5*mer*_), ssm (*de* = 48*, V*_*ssm*_), 1ggap (*de* = 256*, V*_1*ggap*_) and 2ggap (*de* = 256*, V*_2*ggap*_), are extracted from cORFs or noncORFs and are noted as cORFs‐associated feature descriptors, where *de* represents the feature dimension. The feature descriptors quantitatively capture compositional patterns and physicochemical properties, reflecting the distinctive molecular characteristics of the analysed sequences. Detailed information on the descriptors is provided in Supporting Information S1: Table S1.

### 2.4 Overview of Cluster Purity‐Guided Resampling (CluPg)

Conventional machine learning methods typically assume balanced class distributions; however, real‐world datasets often violate this assumption [24]. Such imbalance can bias models towards the majority class, substantially reducing their ability to accurately classify minority class instances [25]. To address this issue, we propose a cluster purity‐guided resampling method, CluPg, specifically designed to mitigate class‐imbalance.

Class‐imbalance in the sample space, from a cluster perspective, decomposes into intracluster imbalance and intercluster overlap, i.e., class‐imbalance within clusters and overlap between clusters. CluPg is a hybrid sampling method that combines undersampling and oversampling phases. In the undersampling phase, the focus is on samples that belong to the same cluster as their neighbours, as these samples are less likely to contribute to class overlap. Conversely, samples whose neighbours are located in different clusters are more prone to overlap, as they are more likely to reside in regions where classes intersect.

To evaluate the distribution of samples within clusters, we introduce the concept of sample cluster purity. The cluster purity $\text{CluP}\left(x_{p}^{c}\right)$ of a sample $x_{p}^{c}$ belonging to cluster *c* is measured by the number $N_{p}^{\text{Difc}}$ of its *k* nearest neighbours that also belong to cluster *c*, providing a quantitative estimate of how well the sample conforms to its assigned cluster, as in the following:

(1) $$\text{CluP}(x_{p}^{c})=\frac{N_{p}^{\text{Difc}}}{k}.$$

Cluster purity $\text{CluP}(x_{p}^{c})$ is calculated for each sample within a cluster. By computing the average cluster purity $\text{Mean}\left(\text{CluP}\left(x^{c}\right)\right)$, the relative position of a sample $x_{p}^{c}$ within its cluster can be determined. If a sample's cluster purity $\text{CluP}(x_{p}^{c})$ is below the average $\text{Mean}\left(\text{CluP}\left(x^{c}\right)\right)$, it is considered to reside in the overlap region and is identified $x_{p}^{c}$ as an overlap sample ${\bar{x}}_{p}^{c}$ within the cluster, as in the following:

(2) $${\bar{x}}_{p}^{c}=\underset{p}{\arg}\left(\text{Clup}(x_{p}^{c})<\text{Mean}\left(\text{Clup}\left(x^{c}\right)\right)\right),$$

Within the sample space, certain samples are initially filtered. To implement more stringent screening, the concept of a dominant cluster is introduced. For a given sample $x_{p}^{c}$, the maximum value of the number of clusters represented among its *k* nearest neighbours is defined as $N_{x_{p}^{c}}^{c}$. The dominant cluster for the sample, PriClus(*x*_*p*_), is then determined based on this value $N_{x_{p}^{c}}^{c}$, providing a reference for further filtering, as in

(3) $$\text{PriClus}\left(x_{p}\right)=\underset{c}{\arg}\max(N_{x_{p}}^{c}).$$

For both majority and minority class samples, if no primary cluster can be identified or multiple primary clusters are detected, the sample is filtered out and excluded from subsequent steps. In this way, overlap samples within the sample space are effectively removed.

During the oversampling phase, new samples are synthesised based on the difference *G* between the sizes of the majority and minority classes after undersampling. The number of samples, *s*_*c*_, to be generated for each cluster is determined according to the proportion of the cluster's sample size difference, *g*_*c*_, relative to the overall difference *G*, as in the following:

(4) $$s_{c}=\frac{1}{\left|c\right|}\times G\times \frac{g_{c}}{\sum_{c}g_{c}},$$

Ultimately, the synthesised sample *x*_sync_ for each cluster is obtained, as in the following:

(5) $$x_{\text{sync}}=x_{p}+\left(x_{p}-x_{q}\right)\;*\lambda ,\lambda \in \left(0,1\right)$$

A critical step involves applying CluPg to preprocess training samples for each feature descriptor independently before model training. As illustrated in Figure 1, CluPg generates synthetic samples specific to each descriptor, resulting in sample distributions that vary across descriptors, a variation that directly affects the performance of the final model ensemble. To address this, we introduce a ‘unified’ sample synthesis approach. In this approach, features from multiple descriptors are first concatenated into a single vector, which is then input to CluPg. The newly synthesised samples generated at this combined level are subsequently partitioned back into their original feature‐level dimensions.

Given that circPEPs are derived from cORFs with coding potential, cORFs‐associated features are incorporated to provide essential biological context. To avoid any dependency on cORFs data during testing, these features are only used during the undersampling stage of CluPg. This strategy allows valuable domain knowledge to inform model training without imposing additional input requirements on the predictive model.

CluPg requires two parameters to be set: the number of clusters *c* and the number of nearest neighbours *k*. In the paper, both *c* and *k* are set to 5.

### 2.5 Fuzzy Voting (FV)

For samples processed through the resampling procedure, model training is conducted using their circPEPs‐associated features. Specifically, each feature descriptor is used to train multiple machine learning models. Although resampling mitigates class‐imbalance in the training–validation set, independent testing sets remain unaltered to preserve the integrity of performance evaluation. A key methodological challenge arises when the distribution of testing samples mirrors that of training samples located in overlap regions, which can skew performance metrics. Samples in these overlap regions often exhibit similar class confidence scores, reducing model robustness. To overcome the limitation of not resampling independent testing sets, we introduce a dynamic ensemble strategy, termed FV, for model fusion. FV adaptively selects base models and adjusts ensemble weights to effectively handle overlapping distributions in the testing data.

This strategy prunes models trained on different feature descriptors, whereas the remaining unfiltered models serve as the base models for ensemble learning. For a sample *x*_*p*_ with the feature descriptor *f*_*i*_, the difference *p*_*ij*_ is calculated based on the confidence probabilities obtained from the base model bm_*j*_, as in.

(6) $$p_{ij}=\left|\text{majc}p_{ij}-\text{minc}p_{ij}\right|$$

where, majcp_*ij*_ and mincp_ij_ denote the confidence probabilities for predicting the majority and minority classes, respectively. For a given sample, the closer the values of majcp_*ij*_ and mincp_*ij*_, the weaker the base model's discriminative ability for that sample. In other words, a smaller *p*_*ij*_ reflects poorer predictive performance of the base model for the corresponding sample. The objective of FV is to adaptively adjust ensemble weights according to the performance of the base models rather than assigning weights manually. During ensemble learning, models with lower *p*_*ij*_ values are assigned smaller weights, whereas a base model bm_*j*_ with a larger *p*_*ij*_ is expected to contribute more substantially to the ensemble and is therefore assigned a higher weight. The original weight *w*_*ij*_ of each model is determined based on its accuracy. By incorporating both *w*_*ij*_ and *p*_*ij*_, the final ensemble weight *e*_*ij*_ is calculated, thereby enabling robust and adaptive fusion of predictions, as in.

(7) $$e_{ij}=\frac{w_{ij}+p_{ij}}{\sum_{j=1}^{m}\left(w_{ij}+p_{ij}\right)}.$$

A predefined threshold *T* is specified. If *e*_*ij*_ is less than or equal to *T*, the corresponding base model *m*_*j*_ is recorded. The number of such base models is calculated using a function denoted as count(). When this number reaches at least half of the total number of base models, the sample is classified as an overlapping sample, ‘t_olp’ and its label *l*_*i*_ is determined as in the following:

(8) $$l=\left\{\begin{array}{l} l_{\text{maj}},\sum_{j=1}^{m}\left(e_{ij}\times \text{maj}p_{ij}\right)\geq \sum_{j=1}^{m}\left(e_{ij}\times \min\;p_{ij}\right)\\ l_{\min},\sum_{j=1}^{m}\left(e_{ij}\times \text{maj}p_{ij}\right)<\sum_{j=1}^{m}\left(e_{ij}\times \min\;p_{ij}\right) \end{array}\right.$$

where *l*_maj_ and *l*_min_ denote the labels of the majority and minority classes, respectively. If the number of base models meeting the threshold does not reach half of the total, the sample is classified as a nonoverlap sample ‘t_nolp’ and its final predicted label is determined using a standard hard voting (HV) strategy.

(9) $$l=\left\{\begin{array}{l} l_{\text{maj}},\text{count}\left(l_{\text{maj}}\right)\geq \text{count}\left(l_{\min}\right)\\ l_{\min},\text{count}\left(l_{\text{maj}}\right)<\text{count}\left(l_{\min}\right) \end{array}\right.,$$

In this study, base models are filtered out if their geometric mean (GM) of sensitivity (SN) and specificity (SP) falls below 85%. The threshold *T* for FV is set to 0.05.

### 2.6 Implementation of CFCPred and Evaluation Criteria

CFCPred is implemented entirely in Python (version 3.9.24). Its predictive performance is evaluated using multiple established metrics, including the Matthews correlation coefficient (MCC), SN, SP, F1‐score (F1) and GM. This multifaceted evaluation provides a comprehensive and robust assessment of the model's classification performance [26].

(10) $$\left\{\begin{array}{l} \text{SP}=\frac{\text{TN}}{\text{TN}+\text{FP}}\\ \text{SN}=\frac{\text{TP}}{\text{TP}+\text{FN}}\\ F1=\frac{2\text{TP}}{2\text{TP}+\text{FP}+\text{FN}}\\ \text{MCC}=\frac{\left(\text{TP}\times \text{TN}\right)-\left(\text{FP}\times \text{FN}\right)}{\sqrt{\left(\text{TP}+\text{FP}\right)\times \left(\text{TP}+\text{FN}\right)\times \left(\text{TN}+\text{FP}\right)\times \left(\text{TN}+\text{FN}\right)}}\\ \text{GM}=\sqrt{\frac{\text{TP}\times \text{TN}}{\left(\text{TN}+\text{FP}\right)\times \left(\text{TP}+\text{FN}\right)}} \end{array}\right.,$$

In the evaluation, true positives (TP) and false negatives (FN) correspond to correctly and incorrectly classified circPEPs (minority class), respectively. True negatives (TN) and false positives (FP) correspond to correctly and incorrectly classified noncircPEPs (majority class), respectively.

## 3 Results

### 3.1 Analysis of CluPg

**FIGURE 2:**
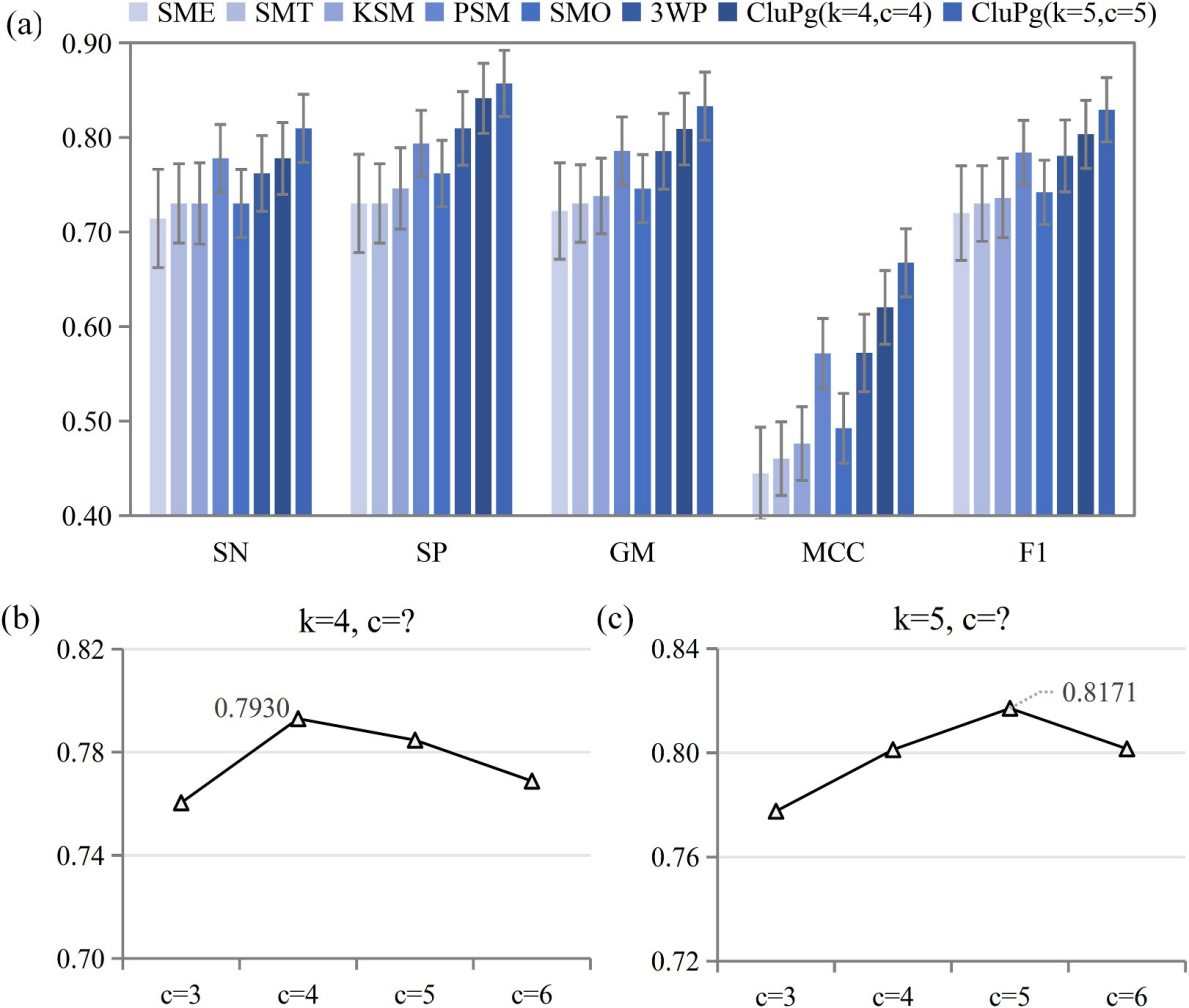
Performance of different resampling techniques. (a) Performance of different resampling techniques on the Dataaset1. (b) GM values of CluPg when *k* = 4 and *c* = ? (c) GM values of CluPg when *k* = 5 and *c* = ?

To address class‐imbalance in the training–validation set, CluPg is applied. For comparative analysis, a set of control groups comprising several classical resampling techniques is established, including SMOTEENN (SME) [27], SMOTETomek (SMT) [28], kmeans‐SMOTE (KSM) [29], PF‐SMOTE (PSM) [30], SMOTE‐OSS (SMO) [31] and 3W‐PEDP (3WP) [32]. All experiments are conducted exclusively on Dataset1, which serves as the basis for resampling, model training and validation. To ensure a standardised evaluation framework, concatenated features of cORFs and circPEPs are separately fed into the undersampling and oversampling phases of each technique. Subsequently, SVM is trained on the balanced data using only the concatenated circPEPs features. Model performance is assessed through five‐fold cross‐validation, generating SN, SP, MCC, F1 and GM metrics. All CluPg operations are performed inside each training fold of the cross‐validation for analysing CluPg. The comparative results are illustrated in Figure 2.

Figure 2a illustrates the performance of CluPg compared with other resampling techniques. The results indicate that CluPg outperforms all alternative methods. The impact of CluPg's two parameters, *k* and *c*, is also evaluated. Figure 2b,c display the results for *k* and *c* is set to 4 or 5. The performance of CluPg at different values of *c*, with *k* set to 3 and 6, is presented in Supporting Information S1: Figure S1. Experimental findings show that setting both *k* and *c* to 5 maximises the GM. Based on these results, CluPg is adopted in CFCPred to balance training samples.

### 3.2 Performance of Machine Learning Models

**FIGURE 3:**
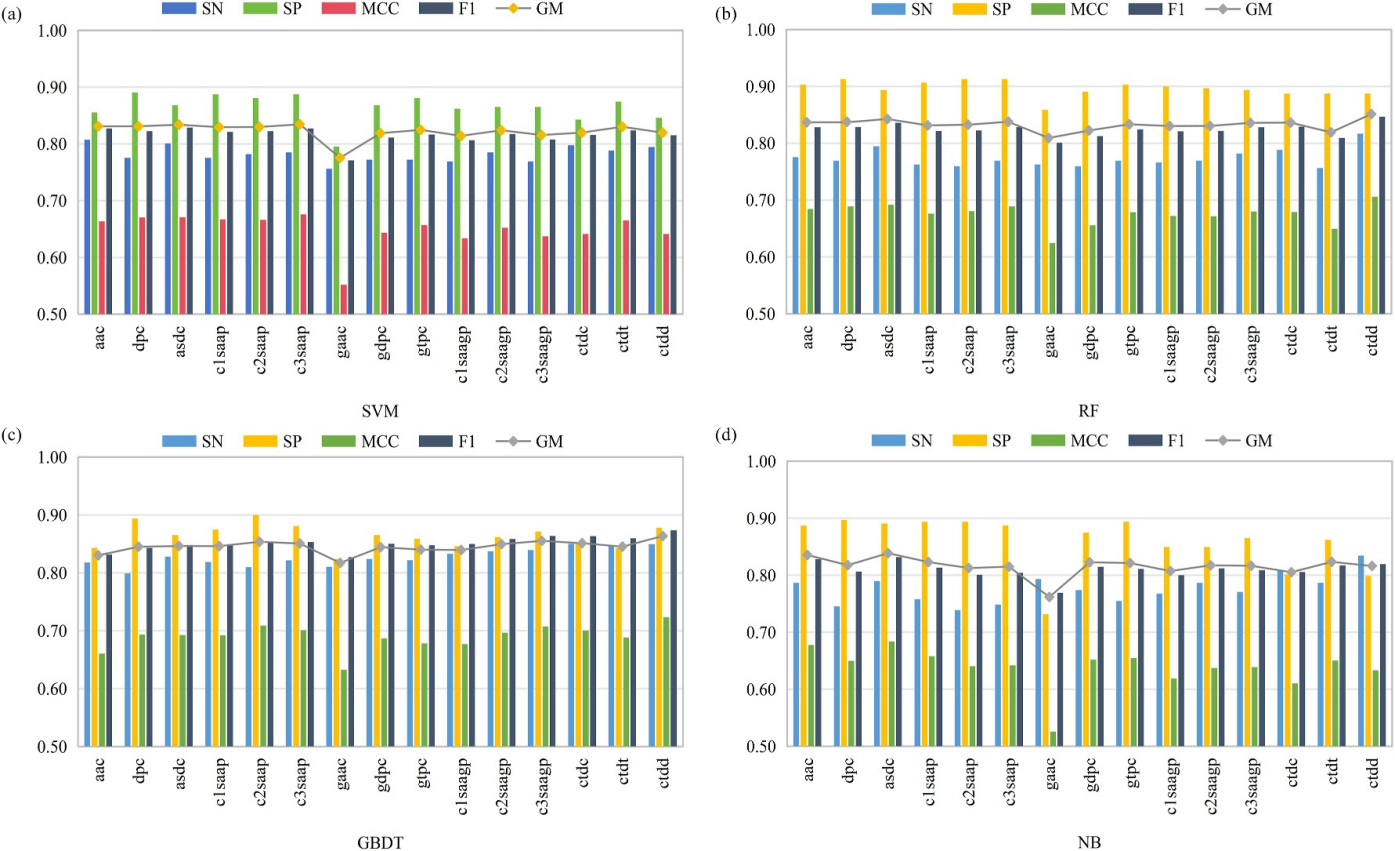
Performance of different machine learning models. (a)–(d) Performance of SVM, RF, GBDT and NB trained with different feature descriptors, separately.

Dataset1, which exhibits class‐imbalance, is used for training and validation. Multiple feature descriptors are extracted, and CluPg is applied to balance the data. The descriptors include AAC, DPC, ASDC, C1SAAP, C2SAAP, C3SAAP, C1SAAGP, C2SAAGP, C3SAAGP, GAAC, GDPC, GTPC, CTDT, CTDD and CTDC. To evaluate the predictive power of different feature descriptors, several machine learning models, including random forest (RF), gradient boosting decision trees (GBDT), Naive Bayes (NB) and SVM, are trained using these features. Five‐fold cross‐validation is implemented, and the SN, SP, PRE, MCC and ACC are recorded for each model. Optimal hyperparameters are provided in Supporting Information S1: Table S2, and the results are summarised in Figure 3.

The experimental results reveal distinct performance patterns across different evaluation metrics. For the minority class, SN, a critical metric, is substantially higher in the tree‐based models, RF and GBDT, compared with NB and SVM. Conversely, SP, which reflects performance on the majority class, is lower in the tree‐based models. When evaluated using composite metrics that balance performance across all samples, including GM, MCC and F1, GBDT consistently outperforms RF, NB and SVM. These results indicate that tree‐based models are particularly effective at identifying minority‐class instances, whereas nontree‐based models perform better on majority‐class classification. This complementary performance profile provides a strong foundation for developing more robust hybrid or ensemble prediction strategies. In addition, feature descriptors such as ASDC, CTDD and C3SAAP demonstrated superior average performance compared with other descriptors. Because each descriptor is used to train different machine learning models, and higher‐performing models are assigned greater weights during model ensembling, these descriptors contributed more substantially to circPEPs prediction. To further investigate the contributions of these descriptors, feature importance analysis is conducted using SHAP values based on the trained random forest model. The results indicate that dipeptide‐level coupling and local sequence order play critical roles in circPEP identification, as shown in Supporting Information S1: Figure S2.

### 3.3 Evaluation of Fuzzy Voting

**FIGURE 4:**
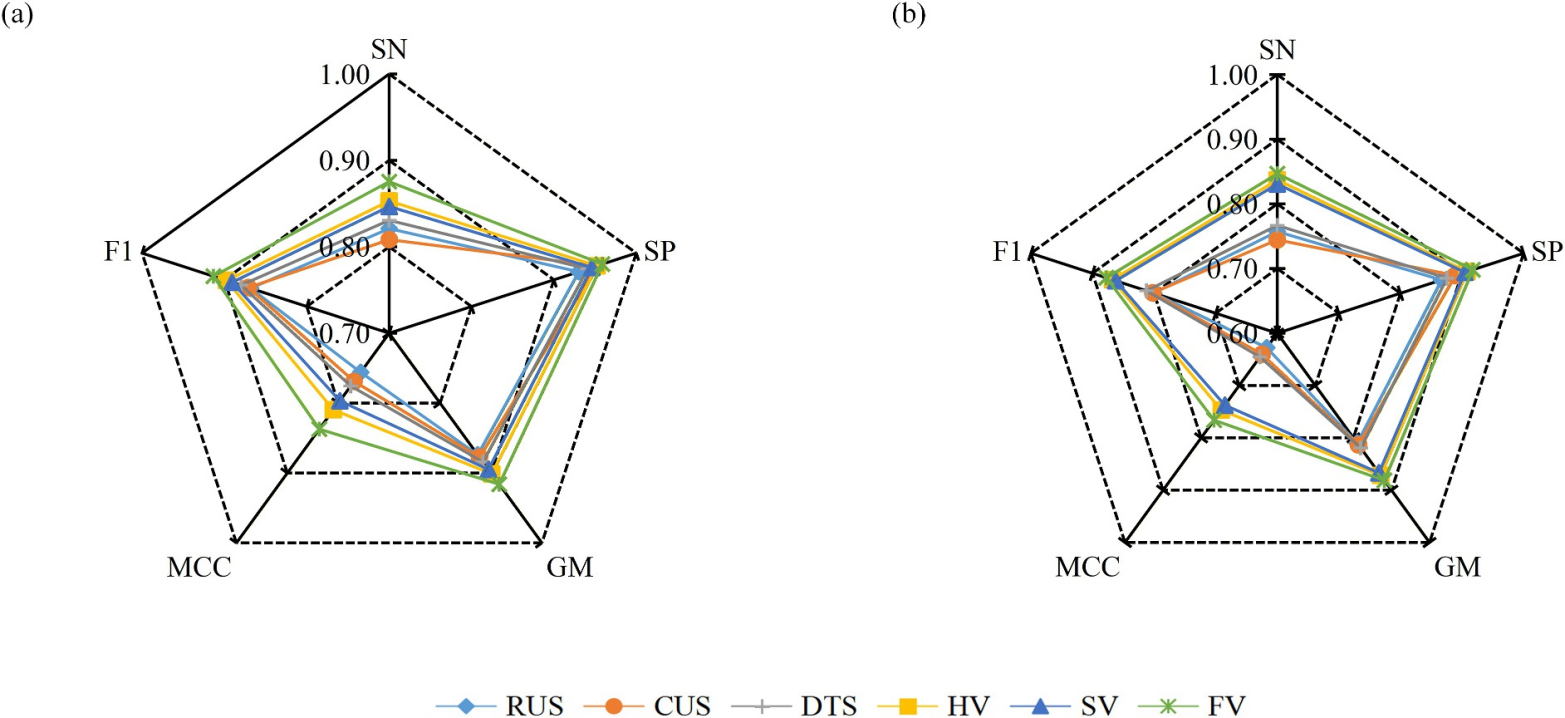
Performance of different ensemble strategies and frameworks. (a) Performance of different ensemble strategies and frameworks in the pruning scenario. (b) Performance of different ensemble strategies and frameworks in the unpruning scenario.

To evaluate the effectiveness of FV, several traditional ensemble strategies, including hard voting (HV) and soft voting (SV), are selected as control groups. Additional control groups are established using existing frameworks that address class‐imbalance through integrated resampling and ensemble strategies, such as RUSBoost (RUS) [33], CUSBoost (CUS) [34] and DTE‐SBD (DTS) [35]. Dataset1 is used for resampling, model training and validation. Since pruning is applied during ensemble construction, each strategy and framework is tested in both pruning and unpruning scenarios. Five‐fold cross‐validation is implemented, and the SN, SP, PRE, MCC and ACC are recorded. The results are presented in Figure 4.

The experimental results demonstrate that FV outperforms other ensemble strategies and frameworks. Compared with the best‐performing strategy in the control group, FV achieved an improvement of approximately 0.006 in overall performance. Furthermore, experiments incorporating pruning (shown in Figure 4a) showed substantially better results than those without pruning (shown in Figure 4b), with an overall performance gain exceeding 0.025.

**FIGURE 5:**
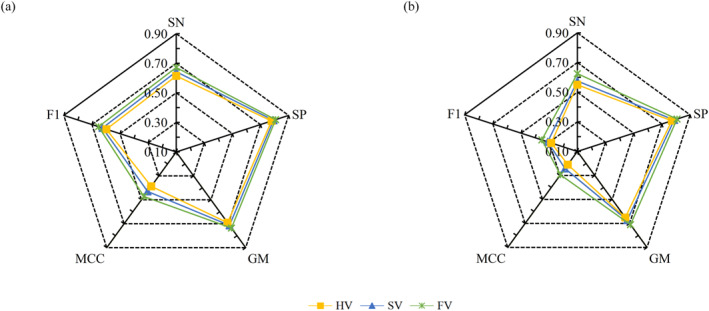
Performance of different voting strategies on the independent‐testing sets. (a) Performance of fuzzy voting (FV), hard voting (HV) and soft voting (SV) on the Dataset2. (b) Performance of fuzzy voting (FV), hard voting (HV) and soft voting (SV) on the Dataset3.

FV is also evaluated on the independent testing sets. HV and SV served as control groups, whereas the rest of the CFCPred architecture remained unchanged. For clarity, the control groups are referred to as CFCPred(hv) and CFCPred(sv), respectively. The results are presented in Figure 5.

The experimental results indicate that FV outperforms other ensemble strategies. Compared with the best‐performing control strategy, FV achieved an improvement of approximately 0.025 in overall performance. Based on these results, FV is adopted in CFCPred to integrate predictions from base models.

### 3.4 Comparison With Other Computational Tools

To evaluate the performance of CFCPred, several existing tools, CPC2 [13], DeepCPP [16], CPESLDI [17], csORFfinder [18], SORFPP [19] and pLM4PEP [20], are used as control groups. Independent testing sets, Dataset2 and Dataset3 are applied are used for evaluation and the best results are highlighted in bold. Because these datasets are class‐imbalanced, MCC, SN, SP, GM and F1‐score are selected as the evaluation metrics. The results are summarised in Table 2.

**TABLE 2:** Performance of tools for predicting circPEPs on the independent‐testing sets.

Tool	Dataset	SN	SP	GM	MCC
CPC2	**Dataset2**	0.6429	0.7143	0.6776	0.3430
DeepCPP		**0.6714**	0.7357	0.7028	0.3918
CPESLDI		0.6143	0.7214	0.6657	0.3244
csORFfinder		0.6286	0.7000	0.6633	0.3150
SORFPP		0.6286	0.7643	0.6931	0.3843
pLM4PEP		0.6429	0.7786	0.7075	0.4134
CFCPred		**0.6714**	**0.8071**	**0.7362**	**0.4723**
CPC2	**Dataset3**	0.5500	0.6425	0.5945	0.1143
DeepCPP		0.6000	0.7250	0.6595	0.2030
CPESLDI		0.5500	0.7125	0.6260	0.1630
csORFfinder		0.5250	0.6900	0.6019	0.1315
SORFPP		0.5750	0.7500	0.6567	0.2082
pLM4PEP		0.5750	0.7750	0.6676	0.2303
CFCPred		**0.6250**	**0.8075**	**0.7104**	**0.2946**

*Note:* Bold values represent the best‐performing score for each metric (SN, SP, GM, MCC) within a given independent‐testing set. Where multiple tools share the identical highest score, all are bolded.

**FIGURE 6:**
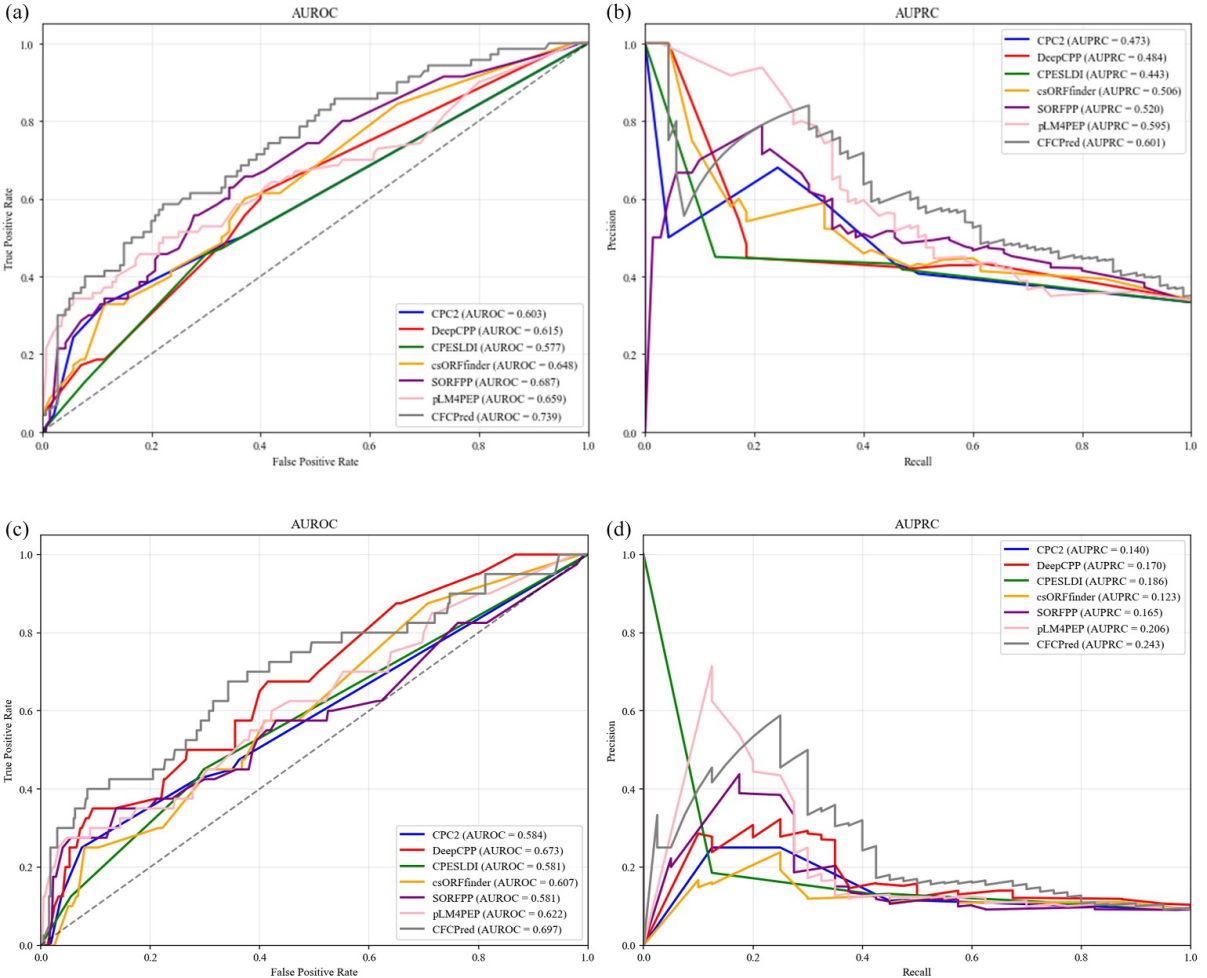
AUPRC and ROC of different tools on the independent‐testing sets. (a)–(b) AUPRC and ROC of CFCPred on the Dataset2. (c)–(d) AUPRC and ROC of CFCPred on the Dataset3.

The experimental results demonstrate that CFCPred outperforms the other computational tools on both Dataset2 and Dataset3. Notably, CFCPred also exhibits superior performance on Dataset2, which contains a larger number of positive samples. Although CPC2, DeepCPP, csORFfinder and CPESLDI can indirectly predict small peptides, they are not specifically designed for circPEPs. The wide range of lengths exhibited by circPEPs results in feature representations that differ from those of typical small peptides, contributing to the superior performance of CFCPred. Additionally, the strong performance of SORFPP and pLM4PEP highlights the promising potential of large language models in peptide prediction. Furthermore, the area under the precision‐recall curve (AUPRC) and receiver operating characteristic (ROC) curves are plotted in Figure 6. CFCPred achieves the highest AUROC and AUPRC values among all evaluated tools, and compared with the best‐performing existing tool, FV provides an additional performance improvement of approximately 0.012.

## 4 Discussion

The comparison with existing tools further validates the effectiveness of multifeature approaches in feature extraction, providing a valuable reference for CFCPred. The multiple features represented in CFCPred embed important domain knowledge. AAC, DPC, ASDC, C1SAAP, C2SAAP, C3SAAP, C1SAAGP, C2SAAGP and C3SAAGP capture sequence composition, reflecting commonalities in amino acid patterns [36], whereas GAAC, GDPC, GTPC, CTDC, CTDT and CTDD encode physicochemical properties within molecules [37]. Leveraging multiple features enables more comprehensive and informative sequence characterisation, which translates into improved model performance. In particular, models trained on features such as AAC, CTDD and ASDC demonstrate superior predictive capabilities. A key advantage of CFCPred is the integration of cORFs‐associated features as prior biological knowledge, enhancing the model's ability to capture relevant signals.

Due to the limited availability of experimentally validated data, bioinformatics tools for rapid and effective circPEPs prediction face significant class‐imbalance challenges. To address this, a novel resampling technology is proposed to balance training samples, mitigating overfitting and overlap issues [26]. The training data used by CPPred, DeepCPP and CPESLDI are also subject to class‐imbalance. Although CPESLDI employs an oversampling method to augment the data, this approach may lead to suboptimal classification performance due to differences between the distributions of the synthesised and original data. In CFCPred, both CluPg and FV are implemented: CluPg addresses class‐imbalance in the training samples prior to model training, whereas FV mitigates the impact of testing samples located in overlap regions. Additionally, SORFPP and pLM4PEP leverage ESM‐2 embeddings [38]. Unlike pLM‐based methods, which typically require high‐end GPUs and substantial memory resources, CFCPred can be executed on standard CPUs. Although pLMs rely on large‐scale pretraining to achieve strong generalisation from limited samples, CFCPred addresses small and imbalanced datasets through advanced sampling strategies. In addition, CFCPred is easier to deploy and offers greater interpretability than pLM‐based embeddings. Although these tools are not specifically developed for circPEP prediction, their strong predictive performance highlights the considerable potential of large language models for peptide prediction tasks [39]. Nevertheless, the limited number of positive samples in the independent testing set represents an inherent limitation of this study. Although Dataset2 includes an increased number of positive samples, future studies should further evaluate the model's generalisation capability using larger and more balanced independent datasets.

The parameters of CluPg are carefully selected: *c*, representing the number of clustering centres, is set to 5 (within the range {3, 4, 5, 6}) and *k*, representing the number of nearest neighbours, is set to 5 (within the range {3, 4, 5, 6}). CluPg refines the positioning of samples beyond the capabilities of basic clustering‐based resampling techniques, laying the foundation for mitigating class‐imbalance. Specifically, during the undersampling and oversampling phase, CluPg introduces the concept of cluster purity to evaluate the distribution of samples within clusters, providing a comprehensive assessment of individual samples, their neighbours and the overall cluster structure. It further characterises the distribution of samples across the entire feature space. Using the notion of dominant clusters, samples located in overlapping regions between different clusters are identified and filtered out. This procedure effectively removes overlapping samples, ensuring that synthetically generated samples during the oversampling stage are minimally distributed in these regions. For each cluster, new samples are synthesised proportionally to mitigate intracluster class imbalance. Furthermore, to evaluate the impact of *c* and *k* on cross‐species prediction, these parameters are systematically varied and CFCPred is assessed on Dataset2 and Dataset3, as shown in Supporting Information S1: Figure S3. The best performance is achieved at *c* = 5 and *k* = 5, although the improvement is marginal.

The threshold *T* is optimised via grid search and ultimately set to 0.05 (selected from {0.02, 0.03, 0.05, 0.07, 0.08}). It is observed that *T* = 0.05 provides the best trade‐off: smaller values introduce excessive low‐confidence votes, whereas larger values discard potentially informative uncertain samples, as shown in Supporting Information S1: Figure S4. The FV strategy is employed to determine the ensemble weights of base models, enabling more accurate predictions. Prior to ensemble construction, pruning is applied to adaptively select base models. On one hand, pruning removes underperforming models, improving overall ensemble performance; on the other hand, it reduces computational runtime by lowering the number of models. FV then calculates ensemble weights based on the confidence probabilities and initial weights of the remaining models. For testing samples, resampling is not applied; samples are directly input into the fixed models. Given the feature distribution, some testing samples may fall within overlap regions of the training data. FV evaluates ensemble weights against a predefined threshold to determine the number of qualifying models. If the number reaches half of the total, the sample is classified as an overlap sample (‘t_olp’), and the ensemble weights are used to determine its final prediction label, with better‐performing models receiving higher weights. If fewer than half of the models meet the threshold, HV alone is used to assign the final label. Experiments show minimal performance improvement for validation samples, largely because the entire training–validation set is preprocessed through CluPg before splitting, indirectly demonstrating CluPg's effectiveness in cleaning overlap samples. Although SV and HV remain viable ensemble approaches [40], FV is preferred here due to its enhanced discriminative capacity for specific sample types.

Moreover, most currently predicted circPEPs are disease‐associated, and experimental results confirm that CFCPred can effectively identify these peptides.

## 5 Conclusions

In this study, we propose CFCPred, a computational framework for predicting circPEPs. CFCPred integrates CluPg and FV within a traditional machine learning pipeline to perform classification tasks. CluPg mitigates class‐imbalance by synthesising samples based on the relationships among individual samples, their nearest neighbours and their cluster assignments. FV addresses overlapping distributions in testing samples by adaptively selecting base models and adjusting ensemble weights. Extensive experimental results demonstrate that CFCPred can accurately and robustly predict circPEPs. Future work will focus on methodological advancements and biological validation. We plan to develop circPEPs‐specific computational frameworks, including tailored large language models, and validate predictions experimentally through targeted assays to establish functional correlations between predicted and observed biological activities.

## Author Contributions

**Siyuan Zhao:** methodology, data curation, writing – original draft. **Bin Yu:** supervision, methodology, writing – original draft, validation. **Lingling Liu:** methodology, writing – reviewing and editing, validation.

## Funding

The authors have nothing to report.

## Conflicts of Interest

The authors declare no conflicts of interest.

## Supporting information


Supporting Information S1

## Data Availability

At https://github.com/zzssyy/CFCPred, the related source is provided on the GitHub repository.
