# Supplementary material for: CFCPred: Advancing circRNAs‐Encoded Peptides Prediction Through Cluster Purity‐Guided Resampling and Fuzzy Voting
Source: IET Syst Biol. 2026 Aug 7;20(1):e70084. doi: 10.1049/syb2.70084 (PMC13449297; doi:10.1049/syb2.70084)
Supplement: Supplementary file 1 [file SYB2-20-e70084-s001.docx]

Table S1 The information of feature descriptors

| **Type** | **Feature Descriptor** | **Definition** | **Dimension** | **Symbolic representation** |
| --- | --- | --- | --- | --- |
| cirPEPs-associated | AAC | Calculate the frequency of single amino acid types in a peptide sequence | 20 | *V_aac_* |
|  | DPC | Calculate the frequency of amino acid pairs in a peptide sequence | 400 | *V_dpc_* |
|  | ASDC | An improved feature descriptor named ASDC based on DPC and CKSAAP | 400 | *V_asdc_* |
|  | CkSAAP (k=1,2,3) | Calculate the frequency of amino acid pairs with the interval of k | 400  400  400 | *V_c_*_1_*_saap_*  *V_c_*_2_*_saap_*  *V_c_*_3_*_saap_* |
|  | CkSAAGP (k=1,2,3) | Calculate the frequency of amino acid  group pairs with the interval of k. | 25  25  25 | *V_c_*_1_*_saagp_*  *V_c_*_2_*_saagp_*  *V_c_*_3_*_saagp_* |
|  | GAAC | Calculate the frequency of each amino acid group (g1: GAVLMI, g2: FYW, g3:KRH, g4:DE, g5:STCPNQ) | 5 | *V_gaac_* |
|  | GDPC | Calculate the frequency of amino acid pairs belonging to different groups | 25 | *V_gdpc_* |
|  | GTPC | Calculate the frequency of 3-tuples amino acid belonging to different groups | 125 | *V_gtpc_* |
|  | CTDC | Calculate the frequency of each amino acid group in a peptide sequence | 39 | *V_ctdc_* |
|  | CTDD | Calculate the fraction of the entire peptide sequence where the first, 25, 50, 75 and 100% of amino acids of a given attribute group that appear in the peptide sequence, respectively | 195 | *V_ctdd_* |
|  | CTDT | Calculate the frequency of adjacent amino acid pairs that the first amino acid belongs to a specific attribute group and the second amino acid belongs to another specific attribute group | 39 | *V_ctdt_* |
| cORFs-associated | k-mer  (k=5) | Use a sliding window of length k to perform sliding matches along with seuqences from beginning to the end with the step of length | 1364 | *V*_5_*_mer_* |
|  | ssm | Calculate the frequency of nonadjacent nucleotide pairs in a sORFs sequence | 48 | *V_ssm_* |
|  | ESkMER  (k=3,4,5) | Enhanced scalable k-mer which integrates both the composition and distance information | 64  256  1024 | *V_es_*_3_*_mer_*  *V_es_*_4_*_mer_*  *V_es_*_5_*_mer_* |
|  | kggap (k=1,2) | Represent the discontinuous k-mer by measuring the correlation of non-adjacent residue pairs | 256  256 | *V*_1_*_ggap_*  *V*_2_*_ggap_* |

Table S2 Details of machine learning models parameter

| **Model** | **Parameter setting** |
| --- | --- |
| NB | GaussianNB() |
| RF | RandomForestClassifier(random_state=100) |
| GBDT | GradientBoostingClassifier(random_state=100) |
| SVM | SVC(random_state=100, probability=True) |

|  | GM |
| --- | --- |
| c=3 | 0.693766 |
| c=4 | 0.726924 |
| c=5 | 0.718421 |
| c=6 | 0.702384 |

|  | GM |
| --- | --- |
| c=3 | 0.745863 |
| c=4 | 0.777616 |
| c=5 | 0.785353 |
| c=6 | 0.7698 |

Fig. S1. (a) GM values for *k*=3, *c*=?. (b) GM values for *k*=6, *c*=?.

|  | times |
| --- | --- |
| dpc | 3 |
| asdc | 8 |
| gdpc | 2 |
| gtpc | 2 |
| ctdd | 13 |
| c1saap | 7 |
| c2saap | 7 |
| c3saap | 8 |

Fig. S2. (a) Feature Contribution Analysis based on SHAP. (b) Number of features in the top50.

(k=?, c=?)

|  | GM | F1 |
| --- | --- | --- |
| (4,4) | 0.688388 | 0.593103 |
| (3,4) | 0.677179 | 0.57931 |
| (6,5) | 0.656444 | 0.554054 |
| (5,5) | 0.736165 | 0.652778 |

(k=?, c=?)

|  | GM | F1 |
| --- | --- | --- |
| (4,4) | 0.687386 | 0.322148 |
| (3,4) | 0.627495 | 0.26087 |
| (6,5) | 0.652878 | 0.289474 |
| (5,5) | 0.710414 | 0.352113 |

Fig. S3. The impact of different *c* and *k* values on the cross-species prediction. (a) on Dataset2. (b) on Dataset3.

T=?

|  | GM | F1 |
| --- | --- | --- |
| 0.02 | 0.638877 | 0.533333 |
| 0.03 | 0.689868 | 0.594595 |
| 0.05 | 0.736165 | 0.652778 |
| 0.07 | 0.691567 | 0.597222 |
| 0.08 | 0.653249 | 0.550336 |

T=?

|  | GM | F1 |
| --- | --- | --- |
| 0.02 | 0.612372 | 0.25 |
| 0.03 | 0.652878 | 0.289474 |
| 0.05 | 0.710414 | 0.352113 |
| 0.07 | 0.672913 | 0.310811 |
| 0.08 | 0.635807 | 0.27451 |

Fig. S4. The impact of different *T* values on the cross-species prediction. (a) on Dataset2. (b) on Dataset3.
